# The Crystal Structure of the Intact *E. coli* RelBE Toxin-Antitoxin Complex Provides the Structural Basis for Conditional Cooperativity

**DOI:** 10.1016/j.str.2012.08.017

**Published:** 2012-10-10

**Authors:** Andreas Bøggild, Nicholas Sofos, Kasper R. Andersen, Ane Feddersen, Ashley D. Easter, Lori A. Passmore, Ditlev E. Brodersen

**Affiliations:** 1 Centre for mRNP Biogenesis and Metabolism, Department of Molecular Biology and Genetics, Aarhus University, DK-8000 Aarhus C, Denmark; 2 MRC Laboratory for Molecular Biology, Cambridge CB2 0QH, UK

## Abstract

The bacterial *relBE* locus encodes a toxin-antitoxin complex in which the toxin, RelE, is capable of cleaving mRNA in the ribosomal A site cotranslationally. The antitoxin, RelB, both binds and inhibits RelE, and regulates transcription through operator binding and conditional cooperativity controlled by RelE. Here, we present the crystal structure of the intact *Escherichia coli* RelB_2_E_2_ complex at 2.8 Å resolution, comprising both the RelB-inhibited RelE and the RelB dimerization domain that binds DNA. RelE and RelB associate into a V-shaped heterotetrameric complex with the ribbon-helix-helix (RHH) dimerization domain at the apex. Our structure supports a model in which *relO* is optimally bound by two adjacent RelB_2_E heterotrimeric units, and is not compatible with concomitant binding of two RelB_2_E_2_ heterotetramers. The results thus provide a firm basis for understanding the model of conditional cooperativity at the molecular level.

## Highlights

► The *E. coli* RelB_2_E_2_ complex has an open V-shaped structure ► Isolated RelE is conformationally flexible ► The structure is not compatible with two copies binding adjacently on DNA ► The structure suggests a model for conditional cooperativity

## Teaser

Bacteria use "self-poisoning" to downregulate cellular processes and to adapt to changing environments. The toxins they use are regulated through tight binding to antitoxins, and Bøggild et al. present the structure of a key intermediate in this subtle regulation mechanism.

## Introduction

The *Escherichia coli relBE* locus encodes a bacterial type II toxin-antitoxin (TA) complex consisting of a toxin, RelE, and its associated antitoxin, RelB (Gerdes et al., 2005). During nutritional stress, the labile RelB is degraded by Lon protease, rendering RelE capable of cleaving messenger RNA (mRNA) during translation on the ribosome, and thus globally downregulating translation (Pedersen et al., 2003). Structural studies of the RelE-ribosome interaction have shown that the toxin employs a reaction mechanism similar to that of bacterial RNase T1, but requires components of the ribosomal RNA in order to properly orient the substrate for endonucleolytic cleavage between nucleotides 2 and 3 of the A-site codon on mRNA (Neubauer et al., 2009). Binding of RelB inhibits RelE by displacement of the C-terminal, flexible α-helix, which contains a tyrosine residue (Tyr87) that is critical for catalysis (Li et al., 2009).

RelB is additionally known to dimerize through formation of a ribbon-helix-helix (RHH) DNA binding motif that allows transcriptional autoregulation through binding to the *relO* operator region (Li et al., 2008; Overgaard et al., 2009). Of interest, it was shown both in vivo and in vitro that transcriptional repression is subtly regulated and depends on the overall RelB:RelE ratio (Overgaard et al., 2008). RelB on its own binds DNA with a relatively low affinity, but binding is dramatically stimulated by addition of RelE up to a RelB:RelE ratio of 2:1. At higher RelE concentrations, the affinity for DNA is lost by a mechanism that has been termed conditional cooperativity (Cataudella et al., 2012; Overgaard et al., 2008, 2009). Mathematical modeling suggests that this system serves two main purposes: (1) to lower the level of free toxin in rapidly growing cells, and (2) to allow cells to return quickly to a low-toxin situation at the end of a starvation period (Cataudella et al., 2012).

The mechanism by which increased amounts of RelE are able to release RelB from DNA is unknown, but the structural basis for conditional cooperativity has been described for another TA locus, *phd/doc* (Garcia-Pino et al., 2010). In this case, the toxin (Doc) contains two antitoxin-binding sites, one with high affinity and one with low affinity. Binding of the antitoxin (Phd) to both sites is required for cooperative DNA binding and transcriptional repression. However, when the relative levels of toxin in the cell increase, the antitoxin molecules eventually will bind only to high-affinity sites, leading to the formation of a Doc-Phd_2_-Doc structure that is not compatible with DNA binding. The presence of high- and low-affinity binding sites for RelB on RelE has been proposed but never experimentally demonstrated (Overgaard et al., 2008). Furthermore, Phd/Doc and RelBE are unrelated in terms of sequence and structure, so a direct functional relationship between them cannot be inferred. Thus, despite numerous structural and functional studies of RelBE, the structural basis for the observed conditional cooperativity, and how transcriptional regulation occurs, remain unclear.

In this work, we describe the crystal structure of the intact *E. coli* RelB_2_E_2_ TA complex determined to 2.8 Å resolution, corresponding to the fully RelE-saturated complex. The structure reveals the spatial arrangement of inhibited RelE relative to the DNA-binding module of RelB and shows that RelB inhibits RelE through a conserved sequence motif that is also found within RelE itself. Two additional crystal structures of isolated RelE further show that the C-terminal helix uses this motif to adopt multiple, defined conformations. Finally, structural superpositioning shows that the distance between binding sites on DNA is incompatible with concomitant binding of two copies of the RelB_2_E_2_ heterotetramer, and therefore provides a structural and mechanistic framework for understanding the phenomenon of conditional cooperativity for the RelBE-type TA loci.

## Results

### RelBE Complex Formation and Structure Determination

Due to translational coupling between the genes of the *relBE* operon, RelB is present in large excess over RelE when the proteins are coexpressed in *E. coli* from a construct representing the natural, genomic context. In order to isolate the fully RelE-saturated RelBE complex, we therefore employed a reciprocal denaturation-renaturation procedure for reconstitution (Overgaard et al., 2009). First, untagged RelE was obtained by on-column denaturation of complex of the R81A active-site mutant and His-tagged RelB, and likewise, untagged RelB was obtained by denaturation of a complex between RelB and His_6_-RelE. As noted previously, isolated RelB behaves as a tetramer in solution, whereas RelE is in a monomeric form (data not shown) (Cherny et al., 2007; Li et al., 2008). Finally, the RelB_2_E_2_ complex was reconstituted by mixing the proteins in the presence of excess RelE to produce the fully RelE-saturated complex.

**Figure 1:**
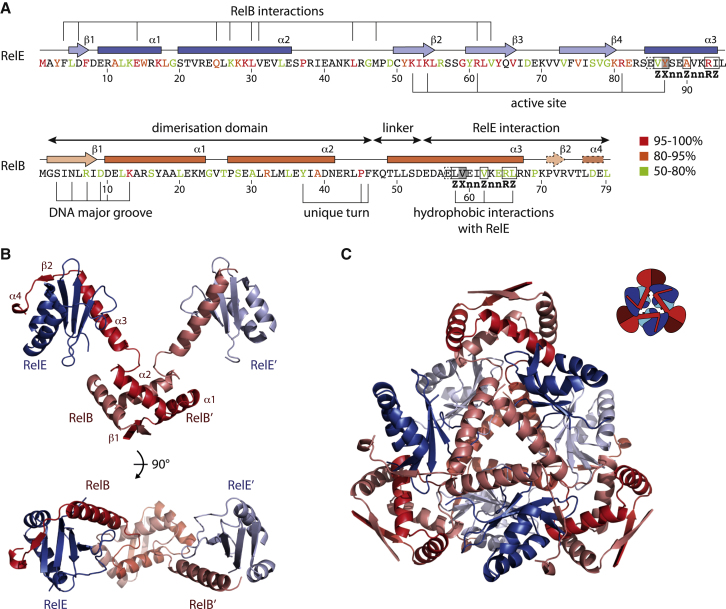
Structural Overview of the *E. coli* RelBE Complex (A) Sequences of *E. coli* RelE and RelB with conservation shown by colored letters as indicated. For RelE, residues that interact with RelB, as well as those involved in catalysis (active site), are shown, and the conserved interaction motif is indicated in bold letters below the corresponding motif. For RelB, individual domains are shown along with residues proposed to interact with the DNA major groove and those that make hydrophobic interactions with RelE. (B) Overview of the RelB_2_E_2_ heterotetramer in two perpendicular views, with RelE in blue and RelB in red. Secondary structure elements in RelB are indicated. (C) The dodecamer assembly observed in the RelBE crystals with an inset showing a simplified overview and colors as in (B). One RelB dimerization domain missing in the structure has been generated by symmetry to show the full assembly. All structure figures were prepared in PyMOL (version 1.3; Schrödinger, L.L.C., http://www.pymol.org). Also see Figure S1.

Large, hexagonal crystals of the complex appeared in 1.9 M (NH_4_)_2_SO_4_ at pH 4.6 and diffracted to 2.8 Å. The crystals belong to space group P6_1_22 with relatively large unit cell dimensions, and structure solution by molecular replacement using the known structures of RelE was not successful. Consequently, the structure was determined by the isomorphous replacement via an anomalous scattering method using multiple heavy atoms (MIRAS), built by iterative model-building, and refined to R (R_free_) = 25.3% (28.5%) (see Supplemental Experimental Procedures available online; Table 1). The final structure comprises three RelBE heterodimers in the crystallographic asymmetric unit (ASU). Two of these heterodimers associate into a heterotetramer by noncrystallographic symmetry, and one engages in a similar interaction through a crystallographic 2-fold axis. The structure covers most of RelE (residues 2–80 of 95) and one molecule of RelB (residues 2–79 of 79), whereas the other two RelB molecules lack either the C terminus (residues 2–69) or N terminus (residues 33–79) due to poor electron density (Figure 1A). The flexible, C-terminal helix of RelE (helix α3, residues 85–95) is disordered in all molecules in this crystal form. We also obtained two different crystal forms of isolated, untagged RelE in 30% w/v PEG 5.000, 0.2 M (NH_4_)_2_SO_4_ at pH 6.5, one of which was previously described (Neubauer et al., 2009). These crystals belong to space group P12_1_1 and contain three molecules per ASU. The other crystal form, belonging to P2_1_2_1_2_1_, contains two molecules per ASU. Both structures were readily determined by molecular replacement using known structures of RelE (PDB 3KHA or 2K29) (Li et al., 2009; Neubauer et al., 2009), and were refined by iterative model building to R (R_free_) = 18.4% (21.9%) for the P12_1_1 form and R (R_free_) = 23.6% (28.2%) for the P2_1_2_1_2_1_ form (Table 1).

### RelB_2_E_2_ Has an Open V-Shaped Structure

In the RelBE complex structure, each RelE tightly binds the C-terminal region of its associated RelB (residues 50–79) through displacement of helix α3 as described previously (Li et al., 2009). Two neighboring RelB molecules dimerize at their N termini to form a RHH-type DNA binding motif that closely resembles the structure of the isolated dimerization domain determined by nuclear magnetic resonance (NMR) (Li et al., 2008). In the resulting heterotetrameric complex, the extended conformation of helix α3 of RelB results in an overall very open, V-shaped complex with an RelE-RelB_2_-RelE architecture and approximate dimensions of 105 × 60 Å (Figure 1B, top). The open structure of the heterotetramer suggests a flexible structure; however, closer inspection of the RelB linker region reveals a unique and stable turn structure centered on Pro45^∗^ (throughout this work, residues in RelB are marked with ^∗^). In the heterotetramer, the two equivalent proline residues are juxtaposed in a symmetrical arrangement that anchors the hydrophobic core of the dimerization domain through interactions between several residues, including Tyr37^∗^, Phe46^∗^, Gln48^∗^, and Arg43^∗^. The functional properties of these residues are quite well conserved among RelB homologs (Figure 1A and Figure S1, purple boxes), so in summary, we believe that the V-shaped structure is relatively rigid and therefore most likely represents the conformation found in vivo.

Inside the crystal, three heterotetramers pack together to form a remarkably compact, nearly spherical superstructure with three RelB dimerization domains at the surface (Figure 1C). This dodecamer has a strikingly large buried surface area of 31.920 Å^2^ (ΔG^int^ = −164.0 kcal/mol), compared with 6.810 Å^2^ (−43.6 kcal/mol) for the RelB_2_E_2_ tetramer as estimated via the Protein Interactions, Surfaces and Assemblies (PISA) server (Krissinel and Henrick, 2007). To assess the potential biological significance of this higher-order structure, we analyzed the RelBE complex in solution by analytical ultracentrifugation (AUC; Table S1) and single-particle cryoelectron microscopy. However, the AUC results show that 96%–99% of the complex is in a tetrameric form in solution, and we were also not able to observe any high-molecular-weight species using cryoelectron microscopy or chemical cross-linking (data not shown). We therefore conclude that the dodecamer is a result of crystal packing and is not representative of the architecture of RelBE in solution.

### Isolated RelE Dimerizes via a Domain-Swap Interaction

**Figure 2:**
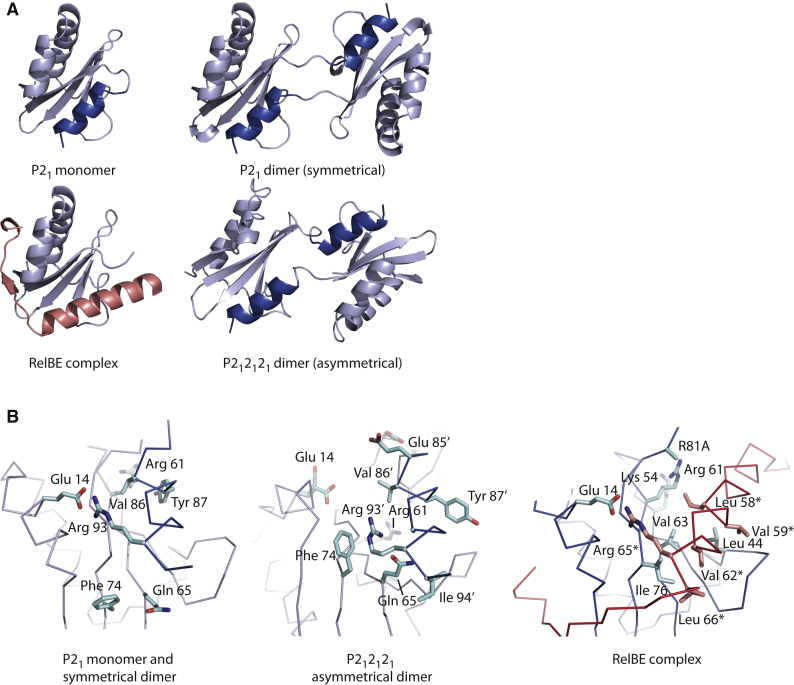
RelE Dimerization and RelB Binding (A) Overview of the RelE structures found in the P2_1_ crystal form (monomer and symmetrical dimer), P2_1_2_1_2_1_ crystal form (asymmetrical dimer), and the RelBE complex. RelE is shown in light blue, with the C-terminal helix α3 in a darker shade. RelB is shown in red. (B) Details of the interactions between the core of RelE and the C-terminal helix (P2_1_ and P2_1_2_1_2_1_ forms) or RelB (in the RelBE structure). Residues from RelE and RelB are shown in blue and red, respectively, with labels marked by an asterisk. Also see Figure S2.

To better assess the structural rearrangements that take place in RelE upon RelB binding, we determined the structures of isolated RelE present in the two crystal forms obtained for the isolated toxin (P2_1_ and P2_1_2_1_2_1_). The structure of the P2_1_ form was previously resolved at 2.5 Å (Neubauer et al., 2009). However, reinvestigation of this structure using better data collected to 1.8 Å revealed that although one of the three molecules in the ASU is indeed in the monomeric form reported earlier (Figure 2A), the two other molecules dimerize via a symmetrical domain-swap interaction involving helix α3. In the structure of the P2_1_2_1_2_1_ crystal form, which has two molecules in the ASU, RelE also forms a domain-swap dimer involving the C-terminal helix; however, this interaction is not symmetrical (Figure 2A). The interaction patterns observed between helix α3 and the core domain are identical in the RelE monomer, symmetrical dimer, and one molecule of the asymmetrical dimer, whereas they differ in the other molecule (Figure 2B). The most common interaction is characterized by a strong salt bridge between Arg93 and Glu14 in addition to multiple contacts between hydrophobic residues such as Val86 and Tyr87 (Figure 2B, left). In the alternative conformation observed in the asymmetrical dimer, the helix is pulled farther back and Arg93 now makes a hydrophobic interaction by stacking its guanidinium group on Phe74 (Figure 2B, middle).

These structures are consistent with NMR studies of RelE and its complex with the interacting helix of RelB, which showed that the helix from RelB lies at an angle of 36° with respect to the orientation of helix α3 in isolated RelE (Li et al., 2009). However, closer analysis reveals that the backbones overlap nearly perfectly at the C-terminal end of the RelB helix, where the interactions are strongest. At the atomic level, the interactions observed here are also surprisingly similar: Arg93 in RelE, which interacts strongly with Glu14 in isolated RelE, superimposes perfectly with Arg65^∗^ from RelB in the complex (Figure 2B, right). Likewise, both Val86 and Ala90 have structurally equivalent residues in RelB (Leu58^∗^ and Val62^∗^; Figure 1A and Figure 2B, right). At the position of Tyr87, which is required for the endonuclease activity of RelE, RelB has a valine (Val59^∗^), thus providing the hydrophobicity while removing the functional group. In summary, there appears to be a consensus motif by which helical interactions with the RelE core occur, which can be expressed as ZXnnZnnRZ (where n is any amino acid, X is a hydrophobic amino acid, and Z is a small hydrophobic amino acid [Val, Ile, Leu, or Ala]). Looking across a wider range of RelE and RelB sequences from various bacteria, this pattern appears to be well conserved (Figure S1).

### A Dimer of RelB_2_E—But Not RelB_2_E_2_—Can Bind to DNA

**Figure 3:**
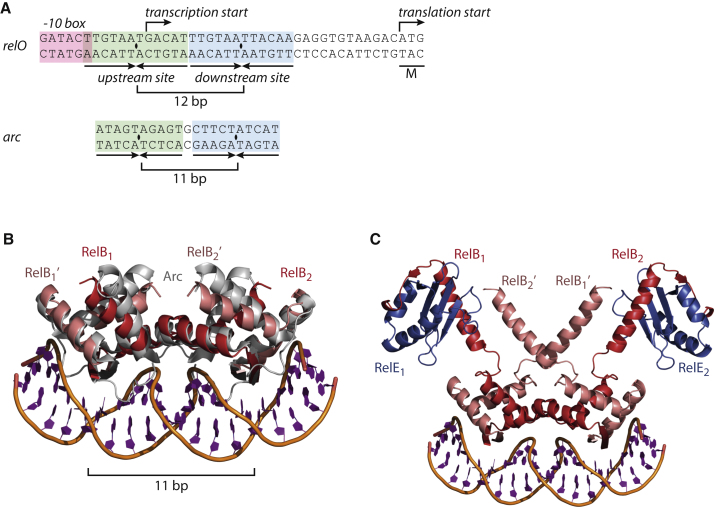
RelB Binds DNA via an Arc-Like Motif (A) Top: The *relO* operator sequence, showing the −10 box (red) and the two sites required for cooperative RelBE binding (green and blue) that overlap with the transcriptional start site (bent arrow). Bottom: The corresponding region of the *arc* operator that provides the binding site for the bacteriophage P22 Arc repressor. (B) Crystal structure of the Arc DNA binding domain (gray) bound to its cognate DNA sequence (orange with bases in purple), with two copies of the RelB dimerization domain overlaid (red). (C) Structural model for binding of two adjacent heterotrimeric RelB_2_E complexes to DNA. Also see Figure S3.

The RelB dimerization domain belongs to the RHH family of DNA binding proteins, which function by inserting two adjacent β-strands into the major groove (Li et al., 2008; Schreiter and Drennan, 2007). A well-described member of this family is the bacteriophage P22 Arc repressor, for which a DNA-bound structure has been determined (PDB ID 1BDT) (Raumann et al., 1994). The *arc* operator consists of two binding sites, each of which has an AT-rich center that allows DNA bending, and has many similarities to the *relO* operator even though it is one basepair shorter (Figure 3A). The crystal structure of the DNA-bound Arc repressor showed that the two sites support binding of a dimer of Arc in each of two adjacent major grooves, and this has been used to create a model for RelB binding to DNA based on the observation that either RelB_2_ or RelB_2_E can bind each leg of the operator (Overgaard et al., 2009; Figure 3B).

Functional studies of operator binding have shown that gradual addition of RelE greatly stimulates DNA binding by RelB at subequimolar quantities but diminishes binding at equimolar ratios, a phenomenon that has been termed conditional cooperativity (Overgaard et al., 2008, 2009). To explain this phenomenon, Overgaard et al. (2008) proposed that RelE harbors two RelB binding sites: a high-affinity binding site used for the catalytic inhibition of RelE, and a low-affinity site required for binding of the complex to DNA in a 1:2 RelE:RelB ratio. This model suggests that when the RelE:RelB ratio increases, RelB will only bind RelE via the high-affinity site and thus release the complex from DNA.

To understand the proposed model in structural terms, we used the structure of DNA-bound Arc to analyze the consequences of binding of the complete RelB_2_E_2_ tetramer to DNA. Structural alignment of two complete RelB_2_E_2_ tetramers with their RelB dimerization domains in adjacent major grooves immediately suggests why the complex cannot bind at high RelE concentrations: Due to the close proximity of the binding sites on DNA, binding of two adjacent tetramers generates an overlapping, W-shaped complex in which the two RelE molecules at each end of the complex (denoted as RelE and RelE′ in Figure S2A) clash. In contrast, if the two most central RelE molecules are removed (corresponding to lowering of the RelE:RelB ratio to 1:2), there are no significant clashes and the four remaining RelB molecules pack accurately together along the axis of the DNA duplex (Figure 3C). Thus, importantly, this architecture permits extensive interactions between all four RelB molecules in the complex, but does not require a secondary low-affinity binding site on RelE. Our structure thus predicts that the RelB_2_E species, which has been shown experimentally to have the highest affinity for DNA, is in fact a RelE-RelB_2_-RelB_2_-RelE W-shaped heterohexameric complex.

## Discussion

In this work, we show that the intact, RelE-saturated RelBE complex from *E. coli* has an unusual V-shaped structure organized by the RelB dimerization domain, and conserved interactions in the loop that connect this domain to the RelE-interaction motif. In this structure, the two RelB-bound RelE molecules are at the distant ends of the V and clash when two RelB dimerization domains bind adjacently on DNA. However, release of one molecule of RelE from each tetramer would allow simultaneous binding of two complexes at the operator site. In contrast to previous models, this structure has the two RelE molecules located on the same side of DNA and is consistent with a tight interaction among all four RelB molecules, thus explaining the observed cooperativity in binding and placing strong restraints on both the distance and angle between the binding sites (Overgaard et al., 2009). Our results thus provide a structural basis for understanding the phenomenon of conditional cooperativity for RelBE-type TA systems at the molecular level.

Furthermore, investigation of other bacterial TA structures reveals that the V shape may be a relatively common architecture that up to now has not been properly appreciated. Methanococcus *jannaschii* RelBE (MjRelBE) contains a minimal RelB interaction domain that is not of the RHH type, yet the overall V-shaped structure is highly reminiscent of the *E. coli* RelB_2_E_2_ complex (Figure S3; Francuski and Saenger, 2009). The structure of RelBE2 from *Mycobacterium tuberculosis* also has a similar architecture, although the long helix in RelB is highly bent (Miallau et al., 2012). Finally, it is noteworthy that the tetrameric structure proposed for Doc-Phd_2_-Doc based on a combination of small-angle X-ray scattering data and a crystal structure of Phd_2_-Doc-Phd_2_ shows a similar arrangement even though the proteins are completely unrelated and have different mechanisms of repression (Figure S3; Garcia-Pino et al., 2010). In summary, we suggest that a V-shaped architecture with the DNA-binding domain at the apex may be a general feature of bacterial TA complexes.

The model of heterohexameric RelE-RelB_4_-RelE allows for a significant number of direct RelB-RelB interactions between the two dimers, both in the RHH domain and along helix α3, thus supporting the observation that pure RelB can form tetramers and bind DNA cooperatively (Cherny et al., 2007; Li et al., 2008). Furthermore, biochemical studies have shown that a RelB fragment covering only residues 1–65 is dimeric. The RelB tetramer model implicates the region around Arg65^∗^, which points toward Asp53^∗^ of the adjacent RelB in the model as being important for the weak cooperativity observed for DNA binding by isolated RelB (Figure S2B; Li et al., 2008; Overgaard et al., 2008). On the other hand, the observation that the RelB construct 1–50 does not show cooperativity implies that interactions between the RHH domains are not critical for this phenomenon (Li et al., 2008).

Although our model does not require a second, low-affinity binding site for RelB on RelE to contribute to cooperativity in the toxin-bound form, it is fully consistent with its existence. Superpositioning of the structure of the C-terminal region of RelB (residues 70–79) from the molecule for which this region is visible onto the innermost RelB molecule of the W indicates that a direct interaction with RelE is possible (Figures S2C and S2D). More precisely, the low-affinity binding site on RelE predicted by this model would consist of the loops between α2/β2 and β2/β3 in RelE and possibly involve interactions with Arg38, Asp49, Lys43, and Glu69. Importantly, however, our structure shows that the presence of a high- and a low-affinity binding site for the antitoxin on the toxin is not a prerequisite for conditional cooperativity, because there are direct RelB-RelB dimer interactions that could potentially be fully responsible for the observed cooperativity if they were stabilized additionally by RelE binding to one RelB molecule. In addition, we note that the architecture of RelBE is different from that of Phd/Doc, in which a Doc toxin molecule bridges two Phd dimers on DNA, leading to the possibility of polymerization as observed by multiple DNA gel shift bands in vitro (Garcia-Pino et al., 2010). In contrast, the closed, W-shaped architecture of the RelBE heterohexamer does not allow for polymerization, which is consistent with the observation that a maximum of two DNA band shifts are observed in vitro (Overgaard et al., 2008).

**Figure 4:**
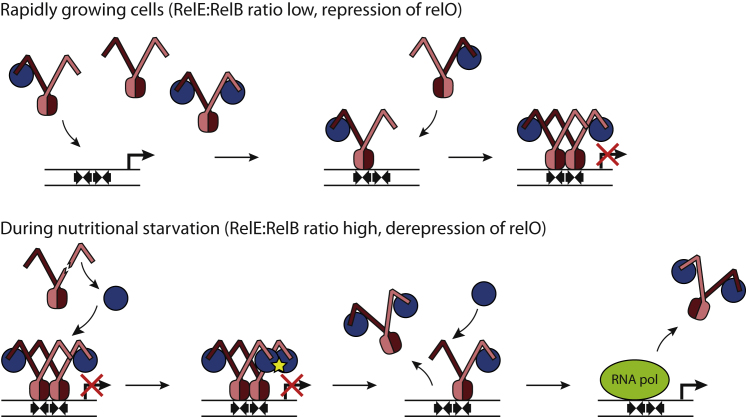
Model for RelBE DNA Binding and Conditional Cooperativity Top: In rapidly growing cells, RelB is in excess and the RelB_2_E trimer initially binds the *relO* operator. Binding of the trimer strongly promotes binding of a second trimer, leading to a RelE-RelB_2_-RelB_2_-RelE heterohexamer binding to the two adjacent sites on DNA and blocking transcription. Bottom: During nutritional starvation, transcription is initially repressed, but the relative amounts of RelE increase as the labile RelB is degraded during translational pausing. Free RelE then binds the unoccupied C-terminal tails of RelB inside the heterohexamer on DNA, leading to a clash and release of the heterotetramer from the DNA. The remaining trimer on DNA also binds a second RelE molecule and is either released through allosteric changes or displaced by the polymerase.

Taken together, our results suggest a model for DNA binding in which during normal, rapid growth, RelB is expressed in excess of RelE, and a mixture of symmetrical RelB_2_E_2_ and RelB_2_ as well as asymmetrical RelB2E complexes will most likely be present, but only the trimer will bind to DNA (Figure 4; Overgaard et al., 2009). Binding of the first trimer strongly promotes binding of an additional complex to the adjacent site on the operator due to favorable interactions between the trimers, eventually causing transcriptional shutdown (Figure 4, top). In contrast, when cells experience nutritional stress and consequently translation slows down, the levels of the labile RelB molecule drop, thus increasing the overall RelE:RelB ratio (Figure 4, bottom). Under these circumstances, an additional RelE molecule will bind to a free RelB C terminus in the heterohexameric complex on the DNA operator, leading to release of RelE-RelB_2_-RelE. This leaves a single RelB_2_E trimer bound to DNA, which will also bind an additional RelE molecule. In this context, we note that DNA band-shift experiments conducted at a high RelE:RelB ratio showed a faint protein-DNA complex that might correspond to a single bound tetramer (Li et al., 2008; Overgaard et al., 2008). Furthermore, surface plasmon resonance measurements revealed that titration of RelE into DNA-bound RelB_2_E led to the formation of a new, stable complex, suggesting that at least in vitro, a single RelB_2_E_2_ (or RelB_2_E) complex may remain bound to the operator even at a very high level of RelE. Finally, it was found that high levels of RelE could not displace RelB_2_E from a single operator site (Overgaard et al., 2008). However, it is likely that the affinity of a single complex for DNA in vivo is too low to prevent polymerase binding and, hence, transcription.

## Experimental Procedures

### Protein Expression and Purification

Untagged RelE^R81A^ was purified by denaturation and refolding from *E. coli* BL21 DE3 (Novagen) harboring a bicistronic construct based on pMG25, expressing both RelE^R81A^ and His-tagged RelB as previously described (Christensen-Dalsgaard et al., 2008; Neubauer et al., 2009). Untagged RelB was purified in a reciprocal way using the plasmid pSC2524HE encoding His-tagged RelE^R81A^ and untagged RelB (Christensen-Dalsgaard et al., 2008). In both cases, the untagged protein was further purified by ion exchange and gel filtration into a final buffer containing 25 mM HEPES, pH 7.0, 100 mM KCl, and 5 mM β-mercaptoethanol (BME; see Supplemental Experimental Procedures for details). Complex formation was achieved by mixing RelE and RelB in the presence of an excess of RelE before final separation by gel filtration and concentration to 9 mg/ml.

### Crystallization and Structure Determination of the RelBE Complex

Hexagonal crystals containing the RelB_2_E_2_ complex grew at 4°C in 1+1 μl sitting-drop vapor diffusion drops with a reservoir of 1.6−2.0 M ammonium sulfate and 0.1 M Na acetate, pH 4.6. Cryoprotection was achieved by gradual transfer of the crystals into 20% glycerol and heavy atom soaks prepared by addition of small amounts of heavy atom salts to the cryo solution. Native data and data from HA-soaked crystals were collected at the MAX-Lab (Lund, Sweden) and processed using XDS (Kabsch, 2010) for the derivative data set and Xia2 (Winter, 2010) for the native set. HA positions were initially located using RANTAN (Collaborative Computational Project, Number 4, 1994), and an improved density-modified MIRAS map was subsequently obtained using only the Pt and Hg derivatives in SHARP (Bricogne et al., 2003). Refinement was carried out by iterative model building in Phenix (Adams et al., 2004) and Coot (Emsley et al., 2010) to a final R (R_free_) of 25.3% (28.5%; see Table 1 for details).

### Crystallization and Structure Determination of Isolated RelE

Full-length *E. coli* RelE^R81A^ was expressed, purified, and crystallized as described previously (Neubauer et al., 2009). Closer inspection of the crystallization drops revealed that they contained two morphologically different, three-dimensional crystal forms, and native data sets were collected from both types. For the previously described crystal form, belonging to the space group P2_1_ (P12_1_1) with three molecules per ASU, improved data extending to 1.8 Å were obtained at beamline ID29 of the European Synchrotron Radiation Facility (ESRF). The other crystal form turned out to belong to space group P2_1_2_1_2_1_ with two RelE molcules per ASU, and for this form, native data were collected at beamline X12 of the European Molecular Biology Laboratory-Deutsches Elektronen Synchrotron (EMBL-DESY) to a maximum resolution of 2.4 Å (Table 1). All data sets were processed using XDS (Kabsch, 2010) or Mosflm via Xia2 (Powell, 1999) and the structures were solved by molecular replacement in Phenix/Phaser (Adams et al., 2004; McCoy et al., 2007) using a search model derived from the published crystal structure of monomeric RelE (PDB ID 3KHA) (Neubauer et al., 2009). From the map generated by Phaser, the models were fitted and rebuilt to include the C-terminal helix by iterative refinement in Phenix and rebuilding in Coot. The final R (R_free_) was 18.4% (21.9%) for the P12_1_1 form and 23.6% (28.2%) for the P2_1_2_1_2_1_ form (see Table 1 and Supplemental Experimental Procedures for details).

### AUC

Purified and reconstituted RelBE complex was analyzed by AUC at a concentration of ∼31 μM using an Optima XL-I analytical ultracentrifuge (Beckmann) at 45,000 rpm and 20°C in 25 mM HEPES, pH 7.0, 150 mM KCl, and 5 mM BME. Data analysis was carried out in Sedfit (Schuck, 2000) using a bimodal distribution of f/f_o_ ratios (see Supplemental Experimental Procedures for details).

## Figures and Tables

**Table 1:** Crystallographic Data and Refinement Statistics

	RelBE (Native)	RelBE (Pt)	RelBE (Hg)	RelE P2_1_ Form	RelE P2_1_2_1_2_1_ Form
**Data Collection**					

Radiation source	MAX-Lab I911-2	MAX-Lab I911-2	Max-Lab I911-2	ESRF ID29	DESY X12
Wavelength (Å)	1.039	1.039	1.039	0.976	0.918

**Data Processing**					

Space group	P6_1_22	P6_1_22	P6_1_22	P2_1_	P2_1_2_1_2_1_
Cell dimensions	a = b = 76.23 Å	a = b = 77.17 Å	a = b = 77.51 Å	a = 42.57 Å	a = 46.63 Å
	c = 362.82 Å	c = 362.79 Å	c = 363.69 Å	b = 61.14 Å	b = 61.44 Å
	α = β = 90°	α = β = 90°	α = β = 90°	c = 70.35 Å	c = 63.90 Å
	γ = 120°	γ = 120°	γ = 120°	α = γ = 90°	α = β = γ = 90°
				β = 102.93°	
Resolution range (Å)	36.3–2.75 (2.82–2.75)	38.59–3.6 (3.69–3.6)	38.76–3.5 (3.59–3.5)	45.6–1.8 (1.85–1.80)	37.7–2.4 (2.46–2.40)
No. of reflections	17,822 (1,249)	14065 (1040)	15,460 (1,139)	32,745 (2,429)	7241 (537)
Completeness (%)	99.8 (99.6)	99.7 (99.0)	99.7 (97.5)	98.0 (97.4)	96.7 (97.8)
Multiplicity	12.9 (13.2)	5.8 (5.0)	6.5 (5.6)	3.7 (3.7)	4.1 (4.1)
Mean I/σ_I_	26.7 (2.7)	8.8 (1.9)	9.2 (2.1)	17.7 (2.2)	20.0 (10.1)
R_sym_ (%)	6.4 (102.3)	18.8 (95.7)	17.1 (92.7)	3.8 (59.9)	4.8 (12.0)
Rmrgd-F (%)	6.7 (60.8)	25.7 (95.8)	21.1 (85.3)	6.7 (76.1)	N/A

**Refinement**					

R_work_ (%)	25.3			18.4	23.6
R_free_ (%)	28.5			21.9	28.2
No. of residues (built/total), solvent, SO_4_-ions	425/516, 21, 3			376/376, 269, 6	182/188, 195, 1
rmsd bond lengths (Å)	0.004			0.006	0.004
rmsd bond angles (degrees)	0.926			1.054	0.827

**Ramachandran Statistics (%)**					

Favored	95.84			95.65	93.26
Allowed	4.16			4.35	6.74
Outliers	0			0	0
PDB deposition ID	4FXE			4FXI	4FXH

Values in parentheses correspond to the outermost resolution shells. See also Table S1.

## Supplemental Information

Document S1. Supplemental Experimental Procedures, Figures S1–S3, and Table S1
