# Supplementary material for: The Crystal Structure of the Intact E. coli RelBE Toxin-Antitoxin Complex Provides the Structural Basis for Conditional Cooperativity
Source: Structure. 2012 Oct 10;20(10):1641–8. doi: 10.1016/j.str.2012.08.017 (PMC3507626; doi:10.1016/j.str.2012.08.017)
Supplement: Document S1. Supplemental Experimental Procedures, Figures S1–S3, and Table S1 [file mmc1.pdf]

Structure, Volume *20*

Supplemental Information

The Crystal Structure of the Intact *E. coli* RelBE Toxin-Antitoxin Complex Provides the Structural Basis for Conditional Cooperativity

Andreas Bøggild, Nicholas Sofos, Kasper R. Andersen, Ane Feddersen, Ashley D. Easter, Lori A. Passmore, and Ditlev E. Brodersen

Inventory of Supplemental Information

Supplemental Experimental Procedures

Figure S1, related to Figure 1. RelE and RelB sequence alignments.

Figure S2, related to Figure 2. Interactions in the higher order complex.

Figure S3, related to Figure 3. Comparison of toxin-antitoxin structures.

Table S1. Analytical ultracentrifugation of reconstituted $RelB_2E_2$ complex

SUPPLEMENTAL EXPERIMENTAL PROCEDURES

Protein expression and purification. Untagged RelE^{R81A} was purified by denaturation from a bicistronic construct based on pMG25 expressing both RelE^{R81A} and His-tagged RelB as previously described (Christensen-Dalsgaard et al., 2008; Neubauer et al., 2009). Untagged RelB was purified in a reciprocal way using the plasmid pSC2524HE encoding His-tagged RelE^{R81A} and untagged RelB (Christensen-Dalsgaard et al., 2008). Both protein complexes were expressed in *E. coli* BL21 DE3 (Novagen) in shaker flasks growing at 37°C by induction with 1mM IPTG for 3 hours. In both cases, harvested cells were opened by sonication in a buffer containing 50 mM Tris-Cl pH 8.0, 300 mM KCl, 5 mM Mg_2Cl_2, 5 mM β-mercaptoethanol (BME), and protease inhibitors followed by high-pressure homogenization. Cleared lysate was loaded onto a 5 mL Ni-column (Qiagen) and washed with lysis buffer containing 20 mM imidazole, before elution of the untagged protein with lysis buffer containing 9 M urea but without Mg_2Cl_2. The denatured elution was shock-refolded by immediate mixing with a 10-fold excess of refolding buffer (50 mM Tris pH 8.0, 5 mM BME, 10% v/v glycerol). For each protein, any aggregated protein was pelleted by centrifugation and the refolded protein further purified using ion exchange at 4°C. RelE was purified on a MonoS column (GE Healthcare) in 0.05 M Hepes pH 7.0, 0.05 – 0.7 M KCl while RelB was purified using a SourceQ column (GE Healthcare) with a 0.05 – 1 M gradient, and both followed by gel filtration on a Superdex 75 10/300 GL (GE Healthcare) column running in 25 mM Hepes, pH 7.0, 100 mM KCl, and 5 mM BME. Protein concentration was estimated using a NanoDrop instrument and extinction coefficients 12,950 $M^{-1}cm^{-1}$ (RelE) and 2,980 $M^{-1}cm^{-1}$ (RelB). The RelE-saturated RelBE complex was then formed by mixing the two proteins in near equimolar amounts but with an excess of RelE and incubating on ice for 1 hour before

final separation on Superdex 75. In this second gel filtration run, the complex elutes at 11.1 mL, while isolated RelB and RelE elute at 10.5 mL and 14.1 mL, respectively.

Crystallisation and structure determination of the RelBE complex. The purified RelBE complex was concentrated to ~9 mg/ml and tested against commercial screens using a Mosquito robot in sitting drop experiments with a 0.2+0.2 µl drop size. Hexagonal crystals containing the $RelB_2E_2$ complex grew at 4°C in 1+1 µL sitting-drop vapour diffusion drops above a reservoir containing 1.6 -2.0 M ammonium sulphate and 0.1 M Na acetate, pH 4.6 using 9.1 mg/mL protein complex. Crystals first appeared after 2 days and grew to maximum dimensions of 150 x 150 x 200 µm within a week. Crystals were cryo-protected in two steps by gradually shifting them from 10 to 20% glycerol before freezing in liquid nitrogen. Heavy atom soaks were prepared by transferring the crystals into cryo-protecting solutions containing small amounts of various heavy atom compounds (Pb, Pt, Hg, Cd, and Yb). Native data and data from HA-soaked crystals were collected at the MAX-Lab (Lund, Sweden) and processed using XDS (Kabsch, 2010) for the derivative dataset and Xia2 (Winter, 2010) for the native. HA positions were initially located using RANTAN, and a phased and an improved density-modified MIRAS map was subsequently obtained using only the Pt and Hg derivatives in SHARP (Bricogne et al., 2003). Refinement was carried out by iterative model building in Phenix (Adams et al., 2004) and Coot (Emsley et al., 2010) to a final R (R_{free}) of 25.3% (28.5%) (see Table 1 for details). The crystal structure has been deposited in the Protein Data Bank with PDB ID 4FXE.

Crystallisation and structure determination of isolated RelE. Full-length *E. coli* $RelE^{R81A}$ was expressed, purified, and crystallised as described previously (Neubauer et al., 2009)□. Closer inspection of the crystallisation drops revealed that they

contained two morphologically different, three dimensional crystal forms and native datasets were collected from both types. For the crystal form previously characterised, belonging to the space group $P2_1$ ($P12_11$) with three molecules per ASU, improved data extending to 1.8 Å were obtained at beam line ID29 at ESRF. The other crystal form turned out to belong to space group $P2_12_12_1$ with two RelE molecules per ASU and for this form, native data were collected at EMBL-DESY X12 to a maximum resolution of 2.4 Å (Table 1). All data sets were processed using XDS (Kabsch, 2010) or MosFlm via Xia2 (Powell, 1999) and the structures were solved by molecular replacement in Phenix/Phaser (Adams et al., 2004; McCoy et al., 2007), using a search model derived from the published crystal structure of monomeric RelE (PDB ID 3KHA) (Neubauer et al., 2009). Side chains were left unchanged in the search model, but the C-terminus from residues 82-95 was removed to minimise model bias when placing the flexible helix in the various RelE copies found the two crystal forms. Based on the map generated by Phaser, the models were fitted and rebuilt to include the C-terminal helix, by iterative refinement in Phenix and rebuilding in Coot. The final R (R_{free}) are 18.4% (21.9%) for the $P12_11$ form and 23.6% (28.2%) for the $P2_12_12_1$ form (see Table 1 for details). Four of five molecules of RelE in the two forms are complete from residue 2 to 95, i.e. just lacking the initial methionine, while molecule A in the $P2_12_12_1$ form lacks residues 55-60 due to poor electron density. For both forms, there was clear extra density at position Cys50, which we have modelled as 2-mercaptoethanol covalently bound to the thiol group of the side chain. The modified positions were included in refinement using eLBOW via ReadySet in Phenix. The final models were submitted to the MolProbity server (molprobity.biochem.duke.edu) for analysis (Davis et al., 2007). Summary statistics for data processing and structure refinement are shown in Table 1. The crystal

structures have been deposited in the Protein Data Bank with PDB ID 4FXI ($P12_11$ form, supersedes the previous entry 3KHA) and 4FXH ($P2_12_12_1$ form).

Analytical ultracentrifugation. Purified and reconstituted RelBE complex was prepared by size exclusion chromatography with multiple runs through a S200 PC 3.2/300 in 25 mM Hepes, pH 7.0, 150 mM KCl, and 5 mM BME and the resulting sample analysed by analytical ultracentrifugation at approximately 31 μM using an Optima XL-I analytical ultracentrifuge (Beckmann). Velocity sedimentation was carried out at 45,000 rpm at 20̊C in 25 mM Hepes, pH 7.0, 150 mM KCl, and 5 mM BME using 12 mm double sector cells in an An60Ti rotor. The sedimentation coefficient distribution function, c(s), was analyzed by the Sedfit program, version 12.51 (Schuck, 2000) using a bimodal distribution of f/f_o ratios. SOMO bead modelling (Rai et al., 2005) predicted frictional ratios of 1.36 and 1.26 for the tetramer and dodecamer, respectively. The ratio for the dodecamer was fixed at 1.26 in the analysis due to the low abundance of species. The solvent density and viscosity (ρ=1.00726 g/ml and η=1.002 mPa·s) were calculated using Sednterp (Dr. Thomas Laue, University of New Hampshire). The major species is a tetramer consistent with a complex of 40.6 kDa with an elongated shape based on a frictional ratio of 1.41 from analysis of the interference data. There is a very low abundance of higher MW species, which is also the reason for the inconsistencies between the absorbance and interference values for these molecules.

SUPPLEMENTAL FIGURES AND TABLES

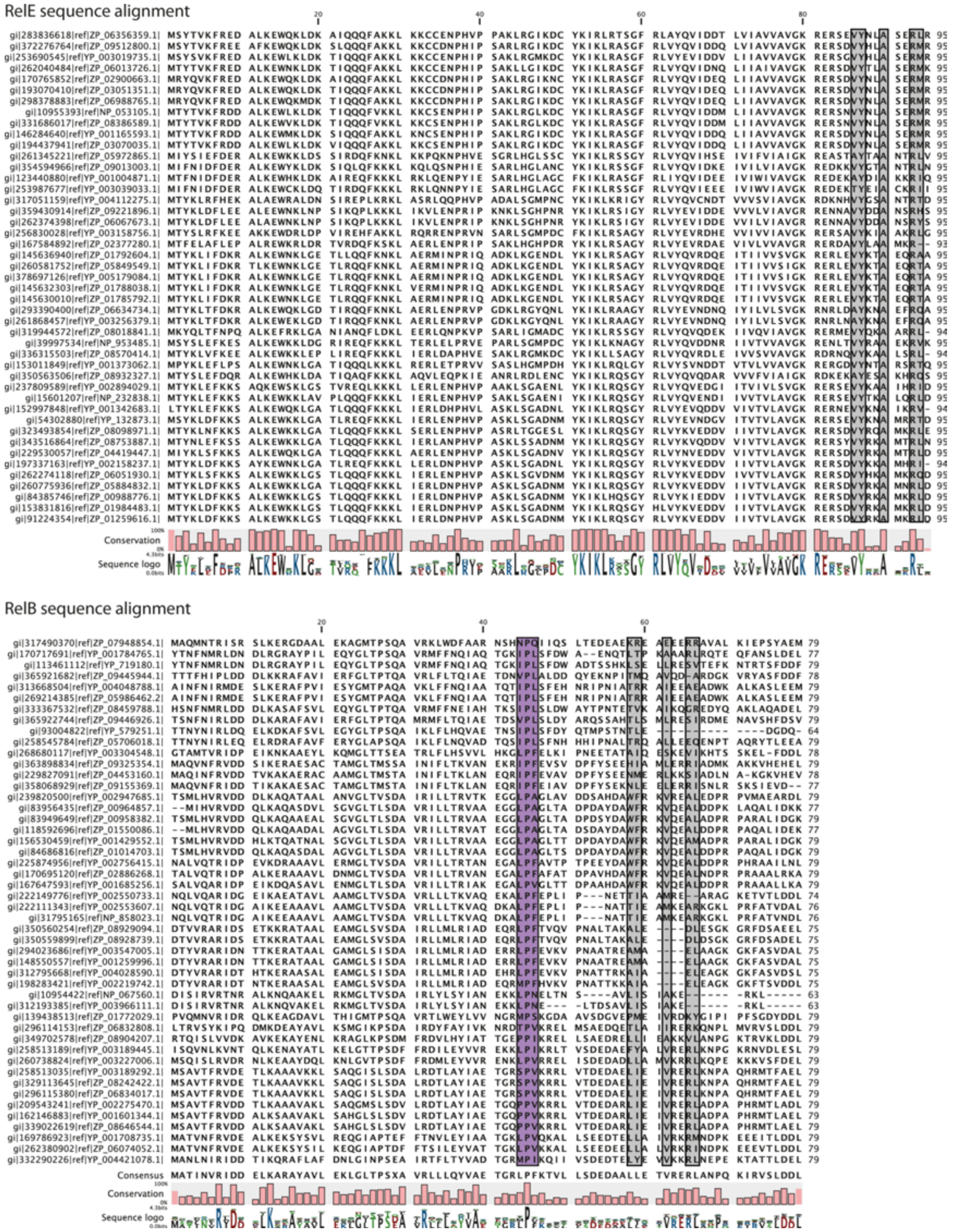

*Supplemental Figure S1, related to Figure 1. RelE and RelB sequence alignments.*Top; alignment of complete RelE sequences from a range of bacterial organisms obtained using PSIBLAST searching with the *E. coli* RelE sequence

(SwissProt ID P0C077). The conserved RelB interaction motif is indicated with grey boxes. Bottom; alignment of complete RelB sequences from a range of bacterial organisms obtained as described above using *E. coli* RelB (SwissProt ID P0C079). The conserved RelE interaction and unique turn motifs are indicated with grey and purple boxes, respectively.

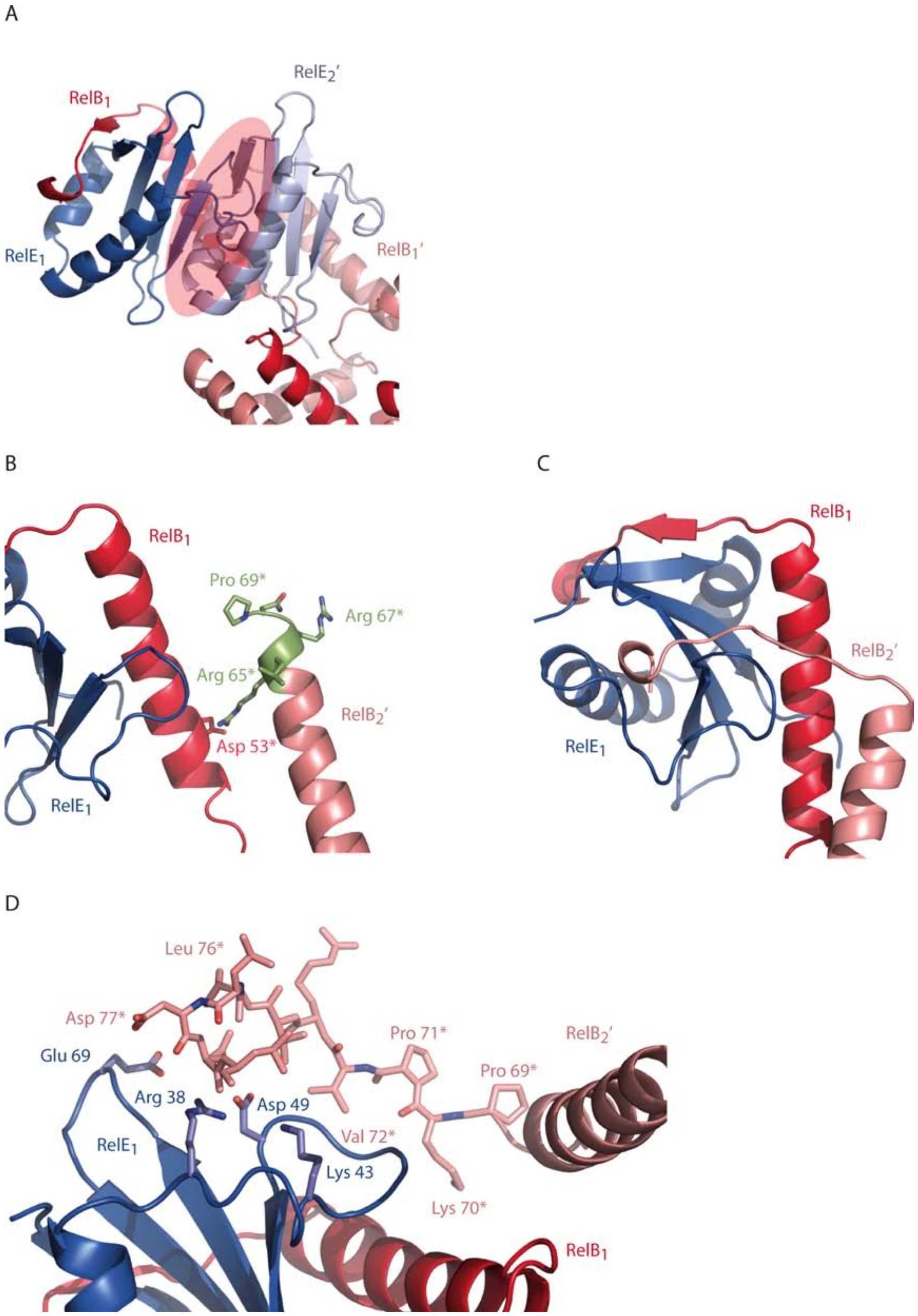

Supplemental Figure S2, related to Figure 2. Interactions in the higher order complex. (A) The clash between RelE molecules upon placement of two complete $RelB_2E_2$ tetramers in adjacent major grooves on DNA. The red oval indicates the area

of clash. (B) Possible interaction between helix $\alpha3$ from two adjacent RelB molecules within the $RelB_4$ tetramer and RelE-$RelB_4$-RelE hexamer. The region 65-69 found to be required for formation of the $RelB_4$ tetramer in solution is coloured in green. (C) Possible location of a secondary binding site for RelB on the surface on RelE as estimated by superpositioning of the most complete RelB molecule onto the innermost RelB molecule of the hexamer model. (D) Details of residues putatively involved in the secondary binding site of RelB on RelE.

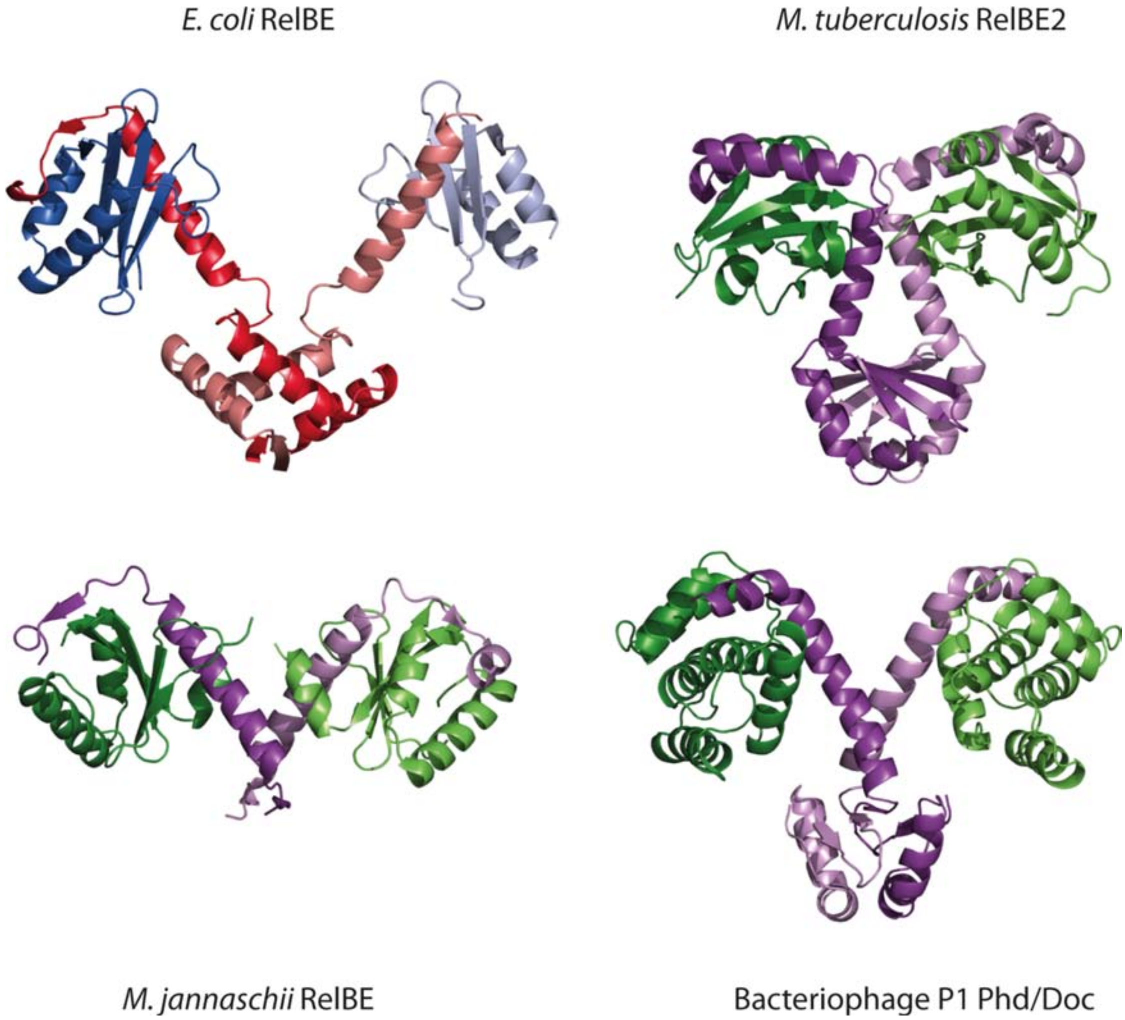

Supplemental Figure S3, related to Figure 3. Comparison of toxin-antitoxin structures. Examples of V-shaped toxin-antitoxin structures. *E. coli* RelBE, this study; *M. tuberculosis* RelBE2, PDB ID 3G5O) (Miallau et al., 2012); M. jannaschii RelBE (PDB ID 3BPQ) (Francuski and Saenger, 2009); bacteriophage P1 Phd/Doc (based on PDB ID 3K33) (Garcia-Pino et al., 2010).

Supplemental Table 1. Analytical ultracentrifugation of reconstituted $RelB_2E_2$ complex.

**Detection method**	**Absorbance**			**Interference**		
**Molecular species**	**1**	**2**	**3**	**1**	**2**	**3**
Fraction (%)	**96**	0.96	1.6	**99.2**	0.07	0.06
MW (kDa)	**40.6**	108.8	231.9	**40.6**	77.8	200
$S_{(20,w)}$	**2.7**	5.9	9.7	**2.7**	4.7	8.8
Frictional ratio	**1.42**	1.26	1.26	**1.41**	1.26	1.26

Supplemental Table 1, related to Table 1. Analytical ultracentrifugation of reconstituted $RelB_2E_2$ complex. Biophysical parameters for the molecular species present in the reconstituted RelBE complex as measured by analytical ultracentrifugation. The predominant species (the $RelB_2E_2$ tetramer) is shown in bold. $S_{(20,w)}$ is the sedimentation coefficient corrected for viscosity and density of the solvent, relative to that of water at 20°C. The frictional ratio is based on SOMO bead modelling. The $RelB_2E_2$ tetramer has a theoretical molecular weight of 40.5 kDa while the $RelB_6E_6$ dodecamer is 108.3 kDa.
